# On a class of integrable Hamiltonian equations in 2+1 dimensions

**DOI:** 10.1098/rspa.2021.0047

**Published:** 2021-05

**Authors:** Ben Gormley, Eugene V. Ferapontov, Vladimir S. Novikov

**Affiliations:** ^1^ Department of Mathematical Sciences, Loughborough University, Loughborough, Leicestershire LE11 3TU, UK; ^2^ Institute of Mathematics, Ufa Federal Research Centre, Russian Academy of Sciences, 112, Chernyshevsky Street, Ufa 450077, Russia

**Keywords:** Hamiltonian PDEs, hydrodynamic reductions, dispersionless Lax pairs, commuting flows, dispersive deformations

## Abstract

We classify integrable Hamiltonian equations of the form

$$u_{t}=\partial_{x}\left(\frac{\delta H}{\delta u}\right),\;H=\int h(u,w)\;\text{d}x\text{d}y,$$

where the Hamiltonian density *h*(*u*, *w*) is a function of two variables: dependent variable *u* and the non-locality $w=\partial_{x}^{-1}\partial_{y}u$. Based on the method of hydrodynamic reductions, the integrability conditions are derived (in the form of an involutive PDE system for the Hamiltonian density *h*). We show that the generic integrable density is expressed in terms of the Weierstrass *σ*-function: *h*(*u*, *w*) = *σ*(*u*) e^*w*^. Dispersionless Lax pairs, commuting flows and dispersive deformations of the resulting equations are also discussed.

## 1. Introduction

### (a) Formulation of the problem

In this paper, we investigate Hamiltonian systems of the form

1.1 $$u_{t}=\partial_{x}\left(\frac{\delta H}{\delta u}\right),\;H=\int h(u,w)\;\text{d}x\text{d}y.$$

Here ∂_*x*_ is the Hamiltonian operator, and the Hamiltonian density *h*(*u*, *w*) depends on *u* and the non-local variable $w=\partial_{x}^{-1}\partial_{y}u$ (equivalently, *w*_*x*_ = *u*_*y*_). Since $\delta H/\delta u=h_{u}+\partial_{x}^{-1}\partial_{y}(h_{w})$ we can rewrite equation (1.1) in the two-component first-order quasi-linear form:

1.2 $$u_{t}={\left(h_{u}\right)}_{x}+{\left(h_{w}\right)}_{y},\;w_{x}=u_{y}.$$

Familiar examples of this type include the dispersionless KP equation (*h* = (1/2)*w*^2^ + (1/6)*u*^3^) and the dispersionless Toda (Boyer–Finley) equation (*h* = e^*w*^). Our main goal is to classify integrable systems within class (1.2) and to construct their dispersionless Lax pairs, commuting flows and dispersive deformations. Before stating our main results, let us begin with a brief description of the existing approaches to dispersionless integrability in 2 + 1 dimensions.

### (b) Equivalent approaches to dispersionless integrability

Here we summarize three existing approaches to integrability of equations of type (1.2), namely, the method of hydrodynamic reductions, the geometric approach based on integrable conformal geometry (Einstein–Weyl geometry) and the method of dispersionless Lax pairs. Based on seemingly different ideas, these approaches lead to equivalent integrability conditions/classification results [1].

*The method of hydrodynamic reductions*, see e.g. [2,3], consists of seeking multi-phase solutions to system (1.2) in the form

1.3 $$u=u(R^{1},R^{2},\ldots ,R^{n})\;\text{and}\;w=w(R^{1},R^{2},\ldots ,R^{n}),$$

where the phases *R*^*i*^(*x*, *y*, *t*) (also known as Riemann invariants; note that their number *n* can be arbitrary) satisfy a pair of commuting hydrodynamic-type systems:

1.4 $$R_{y}^{i}=\mu^{i}(R)R_{x}^{i}\;\text{and}\;R_{t}^{i}=\lambda^{i}(R)R_{x}^{i};$$

we recall that the commutativity conditions are equivalent to the following constraints for the characteristic speeds *μ*^*i*^, *λ*^*i*^ [4,5]:

1.5 $$\frac{\partial_{j}\mu^{i}}{\mu^{j}-\mu^{i}}=\frac{\partial_{j}\lambda^{i}}{\lambda^{j}-\lambda^{i}},$$

$i\neq j,\;\partial_{j}=\partial_{R^{j}}$. Substituting ansatz (1.3) into (1.2) and using (1.4), (1.5) one obtains an overdetermined system for the unknowns *u*, *w*, *μ*^*i*^, *λ*^*i*^, viewed as functions of *R*^1^, …, *R*^*n*^ (the so-called generalized Gibbons–Tsarev system, or GT-system). System (1.2) is said to be integrable by the method of hydrodynamic reductions if it possesses ‘sufficiently many’ multi-phase solutions of type (1.3), in other words, if the corresponding GT-system is involutive. Note that the coefficients of GT-system will depend on the density *h*(*u*, *w*) and its partial derivatives. The requirement that GT-system is involutive imposes differential constraints for the Hamiltonian density *h*, the so-called integrability conditions. Details of this computation will be given in §2a.

*Integrability via Einstein–Weyl geometry.* Let us first introduce a conformal structure defined by the characteristic variety of system (1.2). Given a 2 × 2 quasi-linear system

$$A(v)v_{x^{1}}+B(v)v_{x^{2}}+C(v)v_{x^{3}}=0,$$

where *A*, *B*, *C* are 2 × 2 matrices depending on *v* = (*u*, *w*)^*T*^, the characteristic equation of this system, $\det(Ap_{1}+Bp_{2}+Cp_{3})=0$, defines a conic *g*^*ij*^*p*_*i*_*p*_*j*_ = 0. This gives the characteristic conformal structure [*g*] = *g*_*ij*_*dx*^*i*^*dx*^*j*^, where *g*_*ij*_ is the inverse matrix of *g*^*ij*^. For system (1.2) direct calculation gives

1.6 $$[g]=4h_{ww}\;\text{d}x\text{d}t-\text{d}y^{2}-4h_{uw}\;\text{d}y\text{d}t+4(h_{ww}h_{uu}-h_{uw}^{2})\;\text{d}t^{2};$$

here we set (*x*^1^, *x*^2^, *x*^3^) = (*x*, *y*, *t*). Note that [*g*] depends upon a solution to the system (we will assume [*g*] to be non-degenerate, which is equivalent to the condition *h*_*ww*_ ≠ 0). It turns out that integrability of system (1.2) can be reformulated geometrically as the Einstein–Weyl property of the characteristic conformal structure [*g*]. We recall that Einstein–Weyl geometry is a triple (D,[g],ω), where [*g*] is a conformal structure, D is a symmetric affine connection and *ω* = *ω*_*k*_ d*x*^*k*^ is a one-form such that

1.7 $$D_{k}g_{ij}=\omega_{k}g_{ij}\;\text{and}\;R_{\left(ij\right)}=\Lambda g_{ij}$$

for some function Λ [6,7]; here *R*_(*ij*)_ is the symmetrized Ricci tensor of D. Note that the first part of equations (1.7), known as the Weyl equations, uniquely determines D once [*g*] and *ω* are specified. It was observed in [1] that for broad classes of dispersionless integrable systems (in particular, for systems of type (1.2)), the one-form *ω* is given in terms of [*g*] by a universal explicit formula

$$\omega_{k}=2g_{kj}D_{s}g^{js}+D_{k}\ln\left(\det g_{ij}\right),$$

where $D_{k}$ denotes the total derivative with respect to *x*^*k*^. Applied to [*g*] given by (1.6), this formula implies

1.8 $$\begin{array}{rl} & \omega_{1}=0,\\ & \omega_{2}=\frac{2(h_{uuw}v_{xx}+2h_{uww}v_{xy}+h_{www}v_{yy})}{h_{ww}},\\ & \omega_{3}=\frac{4(h_{uw}(h_{uuw}v_{xx}+2h_{uww}v_{xy}+h_{www}v_{yy}))}{h_{ww}}\\ & \;-\frac{h_{ww}(h_{www}v_{xx}+2h_{uuw}v_{xy}+h_{uww}v_{yy}))}{h_{ww}}. \end{array}\}$$

To summarize, integrability of system (1.2) is equivalent to the Einstein–Weyl property of [g], ω given by (1.6), (1.8) on every solution of system (1.2). Note that in 3 dimensions, Einstein–Weyl equations (1.7) are themselves integrable by the twistor construction [8], see also [9], and thus constitute ‘integrable conformal geometry’.

*Dispersionless Lax pair* of system (1.2) consist of two Hamilton–Jacobi type equations for an auxiliary function *S*,

$$S_{t}=F(S_{x},u,w)\;\text{and}\;S_{y}=G(S_{x},u,w),$$

whose compatibility condition, *S*_*ty*_ = *S*_*yt*_, is equivalent to system (1.2). Dispersionless Lax pairs were introduced in [10] as quasi-classical limits of Lax pairs of integrable soliton equations in 2 + 1 dimensions. It is known that the existence of a dispersionless Lax representation is equivalent to hydrodynamic/geometric integrability discussed above [1,11]. We refer to §2(c) for dispersionless Lax pairs of integrable systems (1.2).

### (c) Summary of the main results

Our first result is the set of integrability conditions for the Hamiltonian density *h*.

**Theorem 1.1.**

*The following conditions are equivalent:*

(a) *System (1.2) is integrable by the method of hydrodynamic reductions;*
(b) *Characteristic conformal structure [g] and covector *ω* given by (1.6), (1.8) satisfy Einstein–Weyl equations (1.7) on every solution of system (1.2);*
(c) *System (1.2) possesses a dispersionless Lax pair;*
(d) *Hamiltonian density *h*(*u*, *w*) satisfies the following set of integrability conditions:*
  1.9 $$\begin{array}{rl} & h_{www}^{2}-h_{ww}h_{wwww}=0,\\ & h_{uww}h_{www}-h_{ww}h_{uwww}=0,\\ & h_{uuw}h_{www}-h_{ww}h_{uuww}=0,\\ & h_{uuu}h_{www}-h_{ww}h_{uuuw}=0\\ & -3h_{uuw}^{2}+4h_{uww}h_{uuu}-h_{ww}h_{uuuu}=0. \end{array}\}$$

Theorem 1.1 is proved in §2a. The system of integrability conditions (1.9) is involutive, and modulo natural equivalence transformations its solutions can be reduced to one of the six canonical forms.

**Theorem 1.2.**

*Solutions* *h*(*u*, *w*) *of system (1.9) can be reduced to one of the six canonical forms:*

$$\begin{array}{rl} & h(u,w)=\frac{1}{2}w^{2}+\frac{1}{6}u^{3},\\ & h(u,w)=w^{2}+u^{2}w-\frac{1}{4}u^{4},\\ & h(u,w)=uw^{2}+\beta u^{7},\\ & h(u,w)={\text{e}}^{w},\\ & h(u,w)=u\;{\text{e}}^{w},\\ & h(u,w)=\sigma (u;0,g_{3})\;{\text{e}}^{w}; \end{array}$$

*here* *β* *and* *g*_3_ *are constants, and* *σ*(*u*;*g*_2_, *g*_3_) *denotes the Weierstrass sigma function.*

Theorem 1.2 is proved in §2b. Dispersionless Lax pairs for the corresponding systems (1.2) are constructed in §2c.

It turns out that every integrable system (1.2) possesses a higher commuting flow of the form

1.10 $$\begin{array}{rl} & u_{\tau }=a(u,w,v)u_{x}+b(u,w,v)u_{y}+c(u,w,v)w_{y}+d(u,w,v)v_{y},\\ & w_{x}=u_{y},\\ & v_{x}=(p{\left(u,w)\right)}_{y}, \end{array}\}$$

where *τ* is the higher ‘time’, and $v=\partial_{x}^{-1}\partial_{y}p(u,w)$ is an extra non-local variable (in contrast to the 1 + 1 dimensional case, higher commuting flows in 2+1 dimensions require higher non-localities). Remarkably, the structure of higher non-localities is uniquely determined by the original system (1.2), in particular, the function *p*(*u*, *w*) can be expressed in terms of *h*(*u*, *w*): *p* = *h*_*w*_. Furthermore, commuting flow (1.10) is automatically Hamiltonian.

**Theorem 1.3.**

*Every integrable system (1.2) possesses a higher commuting flow (1.10) with the non-locality* *v*_*x*_ = (*h*_*w*_)_*y*_. *Commuting flow (1.10) is Hamiltonian with the Hamiltonian density* *f*(*u*, *w*, *v*) *of the form*

$$f(u,w,v)=vh_{w}(u,w)+g(u,w),$$

*where* *g*(*u*, *w*) *can be recovered from the compatible equations*

$$\begin{array}{rl} & g_{ww}=4h_{uw}h_{ww}+\alpha wh_{ww},\\ & g_{uuu}=8h_{uw}h_{uuu}+\alpha wh_{uuu},\\ & g_{uuw}=6h_{uw}h_{uuw}+2h_{ww}h_{uuu}+\alpha wh_{uuw}. \end{array}$$

*Here the constant* *α* *is defined by the relation* *α* = 2*h*_*ww*_ (∂/∂*w*)(*h*_*uw*_/*h*_*ww*_), *which follows from integrability conditions (1.9)*.

Theorem 1.3 is proved in §2d. Dispersionless Lax pairs for commuting flows are constructed in §2e.

## 2. Proofs

In this section, we prove theorems 1.1–1.3 and construct Lax pairs for integrable systems (1.2) and their commuting flows (1.10).

### (a) The method of hydrodynamic reductions: proof of theorem 1.1

**Equivalences (a) ⇔ (b) and (a) ⇔ (c)** of theorem 1.1 follow from the results of [1,11], which hold for general two-component systems of hydrodynamic type in 2+1 dimensions.

**Equivalence (a) ⇔ (d)** can be demonstrated as follows. Let us rewrite system (1.2) in the form

$$u_{t}=h_{uu}u_{x}+2h_{uw}u_{y}+h_{ww}w_{y},\;w_{x}=u_{y},$$

and substitute the ansatz *u* = *u*(*R*^1^, *R*^2^, …, *R*^*n*^), *w* = *w*(*R*^1^, *R*^2^, …, *R*^*n*^). Using equations (1.4) and collecting coefficients at $R_{x}^{i}$ we obtain ∂_*i*_*w* = *μ*^*i*^∂_*i*_*u*, along with the dispersion relation *λ*^*i*^ = *h*_*uu*_ + 2*h*_*uw*_*μ*^*i*^ + *h*_*ww*_(*μ*^*i*^)^2^. Substituting the last formula into the commutativity conditions (1.5), we obtain

2.1 $$\partial_{j}\mu^{i}=\frac{h_{uuu}+h_{uuw}(\mu^{j}+2\mu^{i})+h_{uww}(2\mu^{i}\mu^{j}+{\left(\mu^{i}\right)}^{2})+h_{www}\mu^{j}{\left(\mu^{i}\right)}^{2}}{h_{ww}(\mu^{j}-\mu^{i})}\partial_{j}u.$$

Finally, the compatibility condition ∂_*i*_∂_*j*_*w* = ∂_*j*_∂_*i*_*w* results in

2.2 $$\begin{array}{r} \partial_{i}\partial_{j}u=\frac{2h_{uuu}+3h_{uuw}(\mu^{j}+\mu^{i})+h_{uww}({\left(\mu^{i}\right)}^{2}+4\mu^{i}\mu^{j}+{\left(\mu^{j}\right)}^{2})+h_{www}(\mu^{j}{\left(\mu^{i}\right)}^{2}+\mu^{i}{\left(\mu^{j}\right)}^{2})}{h_{ww}{\left(\mu^{j}-\mu^{i}\right)}^{2}}\partial_{i}u\partial_{j}u. \end{array}$$

Equations (2.1) and (2.2) constitute the corresponding GT-system. As one can see, it contains partial derivatives of the Hamiltonian density *h* in the coefficients. Verifying involutivity of GT-system amounts to checking the compatibility conditions ∂_*k*_(∂_*j*_*μ*^*i*^) = ∂_*j*_(∂_*k*_*μ*^*i*^) and ∂_*k*_(∂_*i*_∂_*j*_*u*) = ∂_*j*_(∂_*i*_∂_*k*_*u*). Direct computation (performed in Mathematica) results in the integrability conditions (1.9) for *h*(*u*, *w*). Note that without any loss of generality one can restrict to the case when the number of Riemann invariants *R*^*i*^ is equal to three, indeed, all compatibility conditions involve three distinct indices only. This finishes the proof of theorem 1.1.

### (b) Canonical forms of integrable densities: proof of theorem 1.2

We have five integrability conditions, namely

2.3 $$\begin{array}{rl} & h_{www}^{2}-h_{ww}h_{wwww}=0, \end{array}$$

2.4 $$\begin{array}{rl} & h_{uww}h_{www}-h_{ww}h_{uwww}=0, \end{array}$$

2.5 $$\begin{array}{rl} & h_{uuw}h_{www}-h_{ww}h_{uuww}=0, \end{array}$$

2.6 $$\begin{array}{rl} & h_{uuu}h_{www}-h_{ww}h_{uuuw}=0 \end{array}$$

2.7 $$\begin{array}{rl} & -3h_{uuw}^{2}+4h_{uww}h_{uuu}-h_{ww}h_{uuuu}=0. \end{array}$$

The classification of solutions will be performed modulo equivalence transformations leaving system (1.2) form-invariant (and therefore preserving the integrability conditions). These include

2.8 $$\tilde{x}=x-2at,\;\tilde{y}=y-2bt,\;\tilde{h}=h+au^{2}+buw+mu+nw+p,$$

as well as

2.9 $$\tilde{x}=x-sy,\;\tilde{w}=w+su;$$

(other variables remain unchanged). We will always assume *h*_*ww*_ ≠ 0, which is equivalent to the requirement of irreducibility of the dispersion relation. There are two main cases to consider.

**Case 1: *h*_*www*_ = 0.** Then

$$h(u,w)=\alpha (u)w^{2}+\beta (u)w+\gamma (u),$$

and the integrability conditions imply

$$\alpha^{\prime \prime }=0,\;\beta^{\prime \prime \prime }=0,\;-3\beta^{\prime \prime 2}+8\alpha^{\prime }\gamma^{\prime \prime \prime }-2\alpha \gamma^{⁗}=0.$$

There are two further subcases: *α* = 1 and *α* = *u*.

The subcase *α* = 1 leads, modulo equivalence transformations (2.8), to densities of the form

$$h(u,w)=w^{2}+\beta_{1}u^{2}w-\frac{\beta_{1}^{2}}{4}u^{4}+\gamma_{1}u^{3},$$

*β*_1_, *γ*_1_ = *const*. For *β*_1_ = 0, we obtain the first case of theorem 1.2 (after a suitable rescaling). If *β*_1_ ≠ 0 then we can eliminate the term *u*^3^ by a translation of *u*. This gives the second case of theorem 1.2 (after rescaling of *u* and *w*).

The subcase *α* = *u* leads, modulo equivalence transformations (2.8), to densities of the form

$$h(u,w)=uw^{2}+\beta_{1}u^{2}w+\gamma_{1}u^{7}+\frac{\beta_{1}^{2}}{4}u^{3}.$$

*β*_1_, *γ*_1_ = const. Note that we can set *β*_1_ = 0 using transformation (2.9) with *s* = *β*_1_/2. This gives the third case of theorem 1.2.

**Case 2: *h*_*www*_ ≠ 0.** Then the first two integrability conditions (2.3) and (2.4) imply *h*_*www*_ = *c h*_*ww*_ for some constant *c* (which can be set equal to 1). This gives

$$h(u,w)=a(u)\;{\text{e}}^{w}+p(u)w+q(u).$$

The next two integrability conditions (2.5) and (2.6) give *p*″ = 0 and *q*″′ = 0, respectively. Thus, modulo equivalence transformations (2.8) we can assume *h*(*u*, *w*) = *a*(*u*) e^*w*^. Finally, equation (2.7) implies

$$aa^{⁗}-4a^{\prime }a^{\prime \prime \prime }+3a^{\prime \prime 2}=0,$$

which is the classical equation for the Weierstrass sigma function (equianharmonic case *g*_2_ = 0). Setting ℘ = −(ln*a*)″ we obtain ℘ ″ = 6℘^2^, which integrates to

2.10 $${℘}^{\prime 2}=4{℘}^{3}-g_{3},$$

*g*_3_ = *const*. There are three subcases.

*Subcase *g*_3_ = 0, ℘ = 0.* Then *a*(*u*) = e^*αu*+*β*^ and modulo equivalence transformations (2.9) we obtain case 4 of theorem 1.2.

*Subcase *g*_3_ = 0, $℘=\frac{1}{u^{2}}$.* Then *a*(*u*) = *u* e^*αu*+*β*^ and modulo equivalence transformations (2.9) we obtain case 5 of theorem 1.2.

*Subcase *g*_3_ ≠ 0.* Then *a*(*u*) = *σ*(*u*;0, *g*_3_) e^*αu*+*β*^ and modulo equivalence transformations (2.9) we obtain the last case of our classification. This finishes the proof of theorem 1.2.

**Remark 1.**

The paper [12] gives a classification of integrable two-component Hamiltonian systems of the form

2.11 $$\left[\begin{array}{c} U_{t}\\ W_{t} \end{array}\right]=\left[\begin{array}{cc} 0 & \partial_{x}\\ \partial_{x} & \partial_{y} \end{array}\right]\left[\begin{array}{c} \frac{\delta H}{\delta U}\\ \frac{\delta H}{\delta W} \end{array}\right],$$

where H=∫F(U,W) dxdy. Explicitly, we have

$$U_{t}={\left(F_{W}\right)}_{x}\;\text{and}\;W_{t}={\left(F_{U}\right)}_{x}+{\left(F_{W}\right)}_{y}.$$

Let us introduce a contact change of variables (*U*, *W*, *F*) → (*u*, *w*, *f*) via partial Legendre transform:

$$w=F_{W},\;u=U,\;f=F-WF_{W},\;f_{w}=-W,\;f_{u}=F_{U}.$$

In the new variables, the system becomes

$$w_{y}=-{\left(f_{u}\right)}_{x}-{\left(f_{w}\right)}_{t},\;u_{t}=w_{x}.$$

Modulo relabelling *u* ↔ *w*, *f* → −*h*, *y* → *t*, *t* → *x*, *x* → *y* these equations coincide with (1.2). Thus, Hamiltonian formalisms (1.1) and (2.11) are equivalent. Examples of dKP and Boyer–Finley equations suggest however that Hamiltonian formalism (1.1) is more natural and convenient, indeed, in the form (1.1) both equations arise directly in their ‘physical’ variables.

### (c) Dispersionless Lax pairs

In this section, we provide dispersionless Lax representations for all six canonical forms of theorem 1.2. The results are summarized in table 1. In the last case, the function *G*(*p*, *u*) is defined by the equations

2.12 $$G_{p}=\frac{G_{uu}}{G_{u}}-\zeta (u)\;\text{and}\;G_{uuu}G_{u}-2G_{uu}^{2}+2℘(u)G_{u}^{2}=0,$$

where *ζ* and ℘ are the Weierstrass functions (equianharmonic case *g*_2_ = 0). The general solution of these equations is given by the formula

2.13 $$G(p,u)=\ln\sigma (\lambda (p-u))+\epsilon \ln\sigma (\lambda (p-\epsilon u))+\epsilon^{2}\ln\sigma (\lambda (p-\epsilon^{2}u)),$$

where $\epsilon ={\text{e}}^{2\pi i/3}=-(1/2)+i(\sqrt{3}/2)$ and $\lambda =i/\sqrt{3}$. Note that the degeneration *g*_3_ → 0, *σ*(*u*) → *u* takes the Lax pair corresponding to the Hamiltonian density *h* = *σ*(*u*) e^*w*^ to the Lax pair for the density *h* = *u* e^*w*^. We refer to the Appendix for a proof that formula (2.13) indeed solves equations (2.12): this requires some non-standard identities for equianharmonic elliptic functions.

**Table 1.** Dispersionless Lax pairs for integrable systems (1.2)

Hamiltonian density *h*(*u*, *w*)	dispersionless Lax pair
$h(u,w)=\frac{1}{2}w^{2}+\frac{1}{6}u^{3}$	
system (1.2):	$S_{t}=\frac{1}{3}S_{x}^{3}+uS_{x}+w$
*u*_*t*_ = *uu*_*x*_ + *w*_*y*_	$S_{y}=\frac{1}{2}S_{x}^{2}+u$
*w*_*x*_ = *u*_*y*_	
$h(u,w)=w^{2}+u^{2}w-\frac{1}{4}u^{4}$	
system (1.2):	$S_{t}=(3u^{2}+2w)S_{x}+2uS_{x}^{4}+\frac{2}{7}S_{x}^{7}$
*u*_*t*_ = (2 *w* − 3*u*^2^)*u*_*x*_ + 4 *u u*_*y*_ + 2*w*_*y*_	$S_{y}=uS_{x}+\frac{1}{4}S_{x}^{4}$
*w*_*x*_ = *u*_*y*_	
*h*(*u*, *w*) = *uw*^2^ + *βu*^7^	
system (1.2):	$S_{t}=4u^{2}℘(S_{x})(w+\frac{1}{5}u^{3}{℘}^{\prime }(S_{x}))$
*u*_*t*_ = 42*βu*^5^*u*_*x*_ + 4*wu*_*y*_ + 2*uw*_*y*_	*S*_*y*_ = *u*^2^℘ (*S*_*x*_)
*w*_*x*_ = *u*_*y*_	here ℘ ′^2^ = 4℘^3^ − 35*β*
*h*(*u*, *w*) = e^*w*^	
system (1.2):	$S_{t}=-\frac{{\text{e}}^{w}}{S_{x}+u}$
*u*_*t*_ = e^*w*^*w*_*y*_	*S*_*y*_ = −ln(*S*_*x*_ + *u*)
*w*_*x*_ = *u*_*y*_	
*h*(*u*, *w*) = *u*e^*w*^	
system (1.2):	$S_{t}=\frac{3u^{2}\;{\text{e}}^{w}S_{x}}{u^{3}-S_{x}^{3}}$
*u*_*t*_ = e^*w*^(2*u*_*y*_ + *uw*_*y*_)	*S*_*y*_ = ln(*S*_*x*_ − *u*) + εln(*S*_*x*_ − ε*u*) + ε^2^ln(*S*_*x*_ − ε^2^ *u*)
*w*_*x*_ = *u*_*y*_	here $\varepsilon =\exp(\frac{2\pi i}{3})$
*h*(*u*, *w*) = *σ*(*u*) e^*w*^	
system (1.2):	*S*_*t*_ = *σ*(*u*) e^*w*^*G*_*u*_(*S*_*x*_, *u*)
*u*_*t*_ = e^*w*^(*σ*″*u*_*x*_ + 2*σ*′*u*_*y*_ + *σw*_*y*_)	*S*_*y*_ = *G*(*S*_*x*_, *u*)
*w*_*x*_ = *u*_*y*_	here *σ*(*u*) = *σ*(*u*;0, *g*_3_)

### (d) Commuting flows: proof of theorem 1.3

Our aim is to show that every integrable system (1.2) possesses a commuting flow of the form (1.10),

$$\begin{array}{rl} & u_{\tau }=a(u,w,v)u_{x}+b(u,w,v)u_{y}+c(u,w,v)w_{y}+d(u,w,v)v_{y},\\ & w_{x}=u_{y}\\ & v_{x}={\left(p(u,w)\right)}_{y}. \end{array}$$

Here *τ* is the higher ‘time’ variable and $v=\partial_{x}^{-1}\partial_{y}p(u,w)$ is a new non-locality (to be determined). Owing to the presence of non-local variables, direct computation of compatibility condition *u*_*tτ*_ = *u*_*τt*_ is not straightforward. Therefore, we adopt a different approach and require that the combined system (1.2) ∪ (1.10),

2.14 $$\begin{array}{rl} & u_{t}={\left(h_{u}\right)}_{x}+{\left(h_{w}\right)}_{y}, \end{array}$$

2.15 $$\begin{array}{rl} & u_{\tau }=a(u,w,v)u_{x}+b(u,w,v)u_{y}+c(u,w,v)w_{y}+d(u,w,v)v_{y}, \end{array}$$

2.16 $$\begin{array}{rl} & w_{x}=u_{y} \end{array}$$

2.17 $$\begin{array}{rl} & v_{x}=(p{\left(u,w)\right)}_{y}, \end{array}$$

possesses hydrodynamic reductions. Thus, we seek multi-phase solutions of the form *u* = *u*(*R*^1^, …, *R*^*n*^), *w* = *w*(*R*^1^, …, *R*^*n*^) and *v* = *v*(*R*^1^, …, *R*^*n*^), where the Riemann invariants *R*^*i*^ satisfy a triple of commuting systems of hydrodynamic type:

$$R_{y}^{i}=\mu^{i}(R)R_{x}^{i},\;R_{t}^{i}=\lambda^{i}(R)R_{x}^{i}\;\text{and}\;R_{\tau }^{i}=\eta^{i}(R)R_{x}^{i}.$$

We recall that the commutativity conditions are equivalent to

2.18 $$\frac{\partial_{j}\mu^{i}}{\mu^{j}-\mu^{i}}=\frac{\partial_{j}\lambda^{i}}{\lambda^{j}-\lambda^{i}}=\frac{\partial_{j}\eta^{i}}{\eta^{j}-\eta^{i}}.$$

Following the same procedure as in §2a, from equations (2.14) and (2.16) we obtain the relations ∂_*i*_*w* = *μ*^*i*^∂_*i*_*u*, the GT-system (2.1), (2.2) and the integrability conditions (1.9) for the Hamiltonian density *h*(*u*, *w*). Similarly, equation (2.17) implies

$$\partial_{i}v=(p_{u}\mu^{i}+p_{w}{\left(\mu^{i}\right)}^{2})\partial_{i}u,$$

and the compatibility condition ∂_*j*_∂_*i*_*v* = ∂_*i*_∂_*j*_*v* results in the relations

$$h_{uuw}p_{w}-h_{ww}p_{uu}=0,\;h_{uww}p_{w}-h_{ww}p_{uw}=0,\;h_{www}p_{w}-h_{ww}p_{ww}=0.$$

Modulo unessential constants of integration (which can be removed by equivalence transformations) these relations uniquely specify the non-locality:

$$p(u,w)=h_{w}(u,w).$$

Finally, equation (2.15) gives an additional dispersion relation,

$$\eta^{i}=a+b\mu^{i}+(c+p_{u}d){\left(\mu^{i}\right)}^{2}+p_{w}d{\left(\mu^{i}\right)}^{3}.$$

Substituting *η*^*i*^ into the commutativity conditions (2.18) we obtain the following set of relations:

2.19 $$\begin{array}{rl} & p_{w}^{2}d_{v}=0, \end{array}$$

2.20 $$\begin{array}{rl} & ((p_{ww}d+p_{w}d_{w})+p_{w}p_{u}d_{v})h_{ww}=2p_{w}dh_{www} \end{array}$$

2.21 $$\begin{array}{rl} & (p_{uw}d+p_{w}d_{u})h_{ww}=2p_{w}dh_{uww}, \end{array}$$

2.22 $$\begin{array}{rl} & h_{ww}(c_{v}+p_{u}d_{v})p_{w}=p_{w}dh_{www}, \end{array}$$

2.23 $$\begin{array}{rl} & h_{ww}((c_{w}+p_{uw}d+p_{u}d_{w})+(c_{v}+p_{u}d_{v})p_{u})=5h_{uww}dp_{w}+ch_{www}+p_{u}dh_{www}, \end{array}$$

2.24 $$\begin{array}{rl} & h_{ww}b_{v}p_{w}=2p_{w}dh_{uww}, \end{array}$$

2.25 $$\begin{array}{rl} & h_{ww}(c_{u}+p_{uu}d+p_{u}d_{u})=4p_{w}dh_{uuw}+ch_{uww}+p_{u}dh_{uww}, \end{array}$$

2.26 $$\begin{array}{rl} & h_{ww}a_{v}p_{w}=p_{w}dh_{uuw}, \end{array}$$

2.27 $$\begin{array}{rl} & h_{ww}(b_{w}+b_{v}p_{u})=4p_{w}dh_{uuw}+2ch_{uww}+2p_{u}dh_{uww}, \end{array}$$

2.28 $$\begin{array}{rl} & h_{ww}b_{u}=2p_{w}dh_{uuu}+2ch_{uuw}+2p_{u}dh_{uuw}, \end{array}$$

2.29 $$\begin{array}{rl} & h_{ww}(a_{w}+a_{v}p_{u})=p_{w}dh_{uuu}+ch_{uuw}+p_{u}dh_{uuw} \end{array}$$

2.30 $$\begin{array}{rl} & h_{ww}a_{u}=ch_{uuu}+p_{u}dh_{uuu}. \end{array}$$

Using the fact that *p* = *h*_*w*_ we solve these relations modulo the integrability conditions (1.9), recall that *h*_*ww*_ ≠ 0. Equation (2.19) gives *d*_*v*_ = 0. Equations (2.20) and (2.21) imply

$$dh_{www}-h_{ww}d_{w}=0,\;dh_{uww}-h_{ww}d_{u}=0,$$

which can be solved for *d*:

$$d=\delta h_{ww},$$

for some constant *δ* (which will be set equal to 2 in what follows). Equation (2.22) gives

$$c_{v}=\text{d}\frac{h_{www}}{h_{ww}}.$$

Setting *c* = d(*h*_*www*_/*h*_*ww*_)*v* + *c*_1_ for some *c*_1_ = *c*_1_(*u*, *w*) and substituting into equation (2.25) we find

$$c_{1}=3dh_{uw}+c_{2}(w)h_{ww}.$$

Substituting *c* = d(*h*_*www*_/*h*_*ww*_)*v* + 3*dh*_*uw*_ + *c*_2_(*w*)*h*_*ww*_ into equation (2.23) we find

$${\left(c_{2}\right)}_{w}=d\left(\frac{h_{uww}h_{ww}-h_{www}h_{uw}}{h_{ww}^{2}}\right)=d\frac{\partial }{\partial w}\left(\frac{h_{uw}}{h_{ww}}\right).$$

It turns out that modulo the integrability conditions (*c*_2_)_*w*_ is a constant. If we set

$$\alpha =\text{d}\frac{\partial }{\partial w}\left(\frac{h_{uw}}{h_{ww}}\right),$$

the final formula for *c* can be written as

$$c=\text{d}\frac{h_{www}}{h_{ww}}v+3dh_{uw}+\alpha wh_{ww}.$$

The equations for the coefficients *a* and *b* cannot be integrated explicitly; rearranging the remaining equations gives the following final result:

$$\begin{array}{rl} & \left\{ \begin{array}{l} a_{u}=\frac{h_{uuu}}{h_{ww}}(c+dh_{uw}),\\ a_{w}=\frac{h_{uuw}}{h_{ww}}c+dh_{uuu},\\ a_{v}=\frac{h_{uuw}}{h_{ww}}. \end{array}\right.\left\{ \begin{array}{l} b_{u}=2(dh_{uuu}+\frac{h_{uuw}}{h_{ww}}(c+dh_{uw})),\\ b_{w}=2(\frac{h_{uww}}{h_{ww}}c+2dh_{uuw}),\\ b_{v}=2d\frac{h_{uww}}{h_{ww}}. \end{array}\right.\\ & c=d\frac{h_{www}}{h_{ww}}v+3dh_{uw}+\alpha wh_{ww},\;d=\delta h_{ww},\;\alpha =d\frac{\partial }{\partial w}\left(\frac{h_{uw}}{h_{ww}}\right). \end{array}$$

The equations for *a* and *b* are consistent modulo integrability conditions (1.9). This proves the existence of commuting flows (1.10).

*Hamiltonian formulation of commuting flows.* Our next goal is to show that the obtained commuting flow can be cast into Hamiltonian form

2.31 $$u_{\tau }=\partial_{x}\left(\frac{\delta F}{\delta u}\right),\;F=\int f(u,w,v)\;\text{d}x\;\text{d}y,$$

with the non-local variables *w*, *v* defined by *w*_*x*_ = *u*_*y*_, *v*_*x*_ = (*h*_*w*_)_*y*_. More precisely, we claim that the commuting density *f* is given by the formula

$$f(u,w,v)=vh_{w}+g(u,w),$$

where the function *g*(*u*, *w*) is yet to be determined. We have

$$\frac{\delta F}{\delta u}=2vh_{uw}+g_{u}+\partial_{x}^{-1}\partial_{y}(2vh_{ww}+g_{w}),$$

so that equation (2.31) takes the form

2.32 $$\begin{array}{rl} & u_{\tau }={\left(2vh_{uw}+g_{u}\right)}_{x}+{\left(2vh_{ww}+g_{w}\right)}_{y},\\ & w_{x}=u_{y},\\ & v_{x}={\left(h_{w}\right)}_{y}. \end{array}\}$$

Explicitly, (2.32) gives

$$\begin{array}{rl} u_{\tau } & =(2vh_{uuw}+g_{uu})u_{x}+(4vh_{uww}+2g_{uw}+2{\left(h_{uw}\right)}^{2})u_{y}\\ & \;+(2h_{uw}h_{ww}+2vh_{www}+g_{ww})w_{y}+2h_{ww}v_{y}. \end{array}$$

Comparing this with (2.15) we thus require

$$\begin{array}{rl} & a=2vh_{uuw}+g_{uu},\\ & b=4vh_{uww}+2g_{uw}+2h_{uw}^{2},\\ & c=2vh_{www}+2h_{uw}h_{ww}+g_{ww},\\ & d=2h_{ww}. \end{array}$$

Using the expressions for *a*, *b*, *c*, *d* calculated above we obtain the equations for *g*(*u*, *w*):

$$\begin{array}{rl} & g_{ww}=4h_{uw}h_{ww}+\alpha wh_{ww},\\ & g_{uuu}=8h_{uw}h_{uuu}+\alpha wh_{uuu}\\ & g_{uuw}=6h_{uw}h_{uuw}+2h_{ww}h_{uuu}+\alpha wh_{uuw}; \end{array}$$

note that these equations are consistent modulo the integrability conditions (1.9). This finishes the proof of theorem 1.3.

### (e) Commuting flows and dispersionless Lax pairs

In this section, we calculate commuting flows of integrable systems (1.2) and construct their dispersionless Lax pairs.

*1. Hamiltonian density *h*(*u*, *w*) = (1/6)*u*^3^ + (1/2)*w*^2^*. The commuting density is

$$f(u,w,v)=vw+u^{2}w.$$

Commuting flow has the form (note that *α* = 0):

$$\begin{array}{rl} & u_{\tau }=2wu_{x}+4uu_{y}+2v_{y},\\ & w_{x}=u_{y}\\ & v_{x}=w_{y}. \end{array}$$

Dispersionless Lax pair:

$$S_{y}=\frac{1}{2}S_{x}^{2}+u$$

$$S_{\tau }=\frac{1}{2}S_{x}^{4}+2uS_{x}^{2}+2wS_{x}+2u^{2}+2v.$$

*2. Hamiltonian density *h*(*u*, *w*) = *w*^2^ + *u*^2^*w* − (1/4)*u*^4^.* The commuting density is

$$f(u,w,v)=2wv+u^{2}v+8uw^{2}-\frac{8}{5}u^{5}.$$

Commuting flow has the form (note that *α* = 0):

$$\begin{array}{rl} & u_{\tau }=(4v-32u^{3})u_{x}+(32w+8u^{2})u_{y}+24uw_{y}+4v_{y},\\ & w_{x}=u_{y},\\ & v_{x}={\left(2w+u^{2}\right)}_{y}. \end{array}$$

Dispersionless Lax pair:

$$\begin{array}{rl} S_{y} & =uS_{x}+\frac{1}{4}S_{x}^{4}\\ S_{\tau } & =(4v+32uw+16u^{3})S_{x}+(8w+24w^{2})S_{x}^{4}+8uS_{x}^{\text{7}}+\frac{4}{5}S_{x}^{10}. \end{array}$$

*3. Hamiltonian density *h*(*u*, *w*) = *uw*^2^ + *βu*^7^.* The commuting density is

$$f(u,w,v)=2uwv+4uw^{3}+20\beta u^{\text{7}}w.$$

Commuting flow has the form (note that *α* = 4):

$$\begin{array}{rl} & u_{\tau }=840\beta u^{5}wu_{x}+(8v+32w^{2}+280\beta u^{6})u_{y}+32uww_{y}+4uv_{y},\\ & w_{x}=u_{y},\\ & v_{x}={\left(2uw\right)}_{y}. \end{array}$$

Dispersionless Lax pair:

$$S_{y}=u^{2}℘(S_{x})$$

$$S_{\tau }=(8u^{2}v+32u^{2}w^{2}+16u^{5}w{℘}^{\prime }(S_{x})+8u^{8}{℘}^{3}(S_{x}))℘(S_{x}),$$

where ℘ ′^2^ = 4℘^3^ − 35*β*.

*4. Hamiltonian density *h*(*u*, *w*) = e^*w*^.* The commuting density is

$$f(u,w,v)=v\;{\text{e}}^{w}.$$

Commuting flow has the form (note that *α* = 0):

$$\begin{array}{rl} & u_{\tau }=2v\;{\text{e}}^{w}w_{y}+2\;{\text{e}}^{w}v_{y},\\ & w_{x}=u_{y}\\ & v_{x}={\left({\text{e}}^{w}\right)}_{y}. \end{array}$$

Dispersionless Lax pair:

$$S_{y}=-\ln(S_{x}+u)$$

$$S_{\tau }=\frac{-2v\;{\text{e}}^{w}}{S_{x}+u}+\frac{{\text{e}}^{2w}}{{\left(S_{x}+u\right)}^{2}}.$$

*5. Hamiltonian density *h*(*u*, *w*) = *u* e^*w*^.* The commuting density is

$$f(u,w,v)=uv\;{\text{e}}^{w}+u\;{\text{e}}^{2w}.$$

Commuting flow has the form (note that *α* = 0):

$$\begin{array}{rl} & u_{\tau }=(4v\;{\text{e}}^{w}+6\;{\text{e}}^{2w})u_{y}+(2uv\;{\text{e}}^{w}+6u\;{\text{e}}^{2w})w_{y}+2u\;{\text{e}}^{w}v_{y},\\ & w_{x}=u_{y},\\ & v_{x}={\left(u\;{\text{e}}^{w}\right)}_{y}. \end{array}$$

Dispersionless Lax pair:

$$S_{y}=\ln(S_{x}-u)+\varepsilon \ln(S_{x}-\varepsilon u)+\varepsilon^{2}\ln(S_{x}-\varepsilon^{2}u)$$

$$S_{\tau }=\frac{3u^{2}\;{\text{e}}^{w}S_{x}(2u^{3}v-2vS_{x}^{3}-3\;{\text{e}}^{w}S_{x}^{3})}{{\left(S_{x}^{3}-u^{3}\right)}^{2}}.$$

*6. Hamiltonian density *h*(*u*, *w*) = *σ*(*u*) e^*w*^.* The commuting density is

$$f(u,w,v)=v\sigma (u)\;{\text{e}}^{w}+\sigma (u)\sigma^{\prime }(u)\;{\text{e}}^{2w}.$$

Commuting flow has the form (note that *α* = 0):

$$\begin{array}{rl} u_{\tau } & =(2v\sigma^{\prime \prime }\;{\text{e}}^{w}+{\left(\sigma \sigma^{\prime }\right)}^{\prime \prime }\;{\text{e}}^{2w})u_{x}+(4v\sigma^{\prime }\;{\text{e}}^{w}+(4\sigma \sigma^{\prime \prime }+6\sigma^{\prime 2})\;{\text{e}}^{2w})u_{y}\\ & \;+(2v\sigma \;{\text{e}}^{w}+6\sigma \sigma^{\prime }\;{\text{e}}^{2w})w_{y}+2\sigma \;{\text{e}}^{w}v_{y},\\ w_{x} & =u_{y}\\ v_{x} & ={\left(\sigma \;{\text{e}}^{w}\right)}_{y}. \end{array}$$

Dispersionless Lax pair:

$$S_{y}=G(S_{x},u)$$

$$S_{\tau }=2[v\;{\text{e}}^{w}\sigma (u)+\;{\text{e}}^{2w}\sigma (u)\sigma^{\prime }(u)]G_{u}(S_{x},u)-{\text{e}}^{2w}\sigma {\left(u\right)}^{2}G_{uu}(S_{x},u),$$

here *G*(*S*_*x*_, *u*) is defined by equations (2.12).

## 3. Dispersive deformations

Dispersive deformations of hydrodynamic type systems in 1 + 1 dimensions were thoroughly investigated in [13–16] based on deformations of the corresponding hydrodynamic symmetries. In 2 + 1 dimensions, an alternative approach based on deformations of hydrodynamic reductions was proposed in [17,18].

It still remains a challenging problem to construct dispersive deformations of all Hamiltonian systems (1.2) obtained in this paper (we do not know whether these deformations always exist as formal series in the deformation parameter *ϵ* whose coefficients are differential polynomials in *u* and *w*, as well as whether additional higher non-localities will be required). In general, all three ingredients of the construction may need to be deformed, namely, the Hamiltonian operator ∂_*x*_, the Hamiltonian density *h*(*u*, *w*) and the non-locality *w*. Here we give just two examples.

**Example 3.1 (dKP equation).**

The Hamiltonian density *h* = (1/2)*w*^2^ + (1/6)*u*^3^ results in the dKP equation:

$$u_{t}=uu_{x}+w_{y},\;w_{x}=u_{y}.$$

It possesses an integrable dispersive deformation

$$u_{t}=uu_{x}+w_{y}-\epsilon^{2}u_{xxx},\;w_{x}=u_{y},$$

which is the full KP equation (in this section *ϵ* denotes an arbitrary deformation parameter). The KP equation corresponds to the deformed Hamiltonian density

$$h(u,w)=\frac{1}{2}w^{2}+\frac{1}{6}u^{3}+\frac{\epsilon^{2}}{2}u_{x}^{2},$$

while the Hamiltonian operator ∂_*x*_ and the non-locality $w=\partial_{x}^{-1}\partial_{y}u$ stay the same. Indeed, we have

$$u_{t}=\partial_{x}\frac{\delta H}{\delta u}=\partial_{x}(\partial_{x}^{-1}\partial_{y}w+\frac{1}{2}u^{2}-\epsilon^{2}u_{xx})=uu_{x}+w_{y}-\epsilon^{2}u_{xxx}.$$

**Example 3.2 (Boyer–Finley equation).**

The Hamiltonian density *h* = e^*w*^ results in the dispersionless Toda (Boyer–Finley) equation:

$$u_{t}={\text{e}}^{w}w_{y},\;w_{x}=u_{y}.$$

It possesses an integrable dispersive deformation

$$u_{t}=\left(\frac{1-T^{-1}}{\epsilon }\right){\text{e}}^{w},\;w_{x}=\left(\frac{T-1}{\epsilon }\right)u,$$

which is the full Toda equation. Here *T* and *T*^−1^ denote the forward/backward *ϵ*-shifts in the *y*-direction, so that $\frac{T-1}{\epsilon }$ and $\frac{1-T^{-1}}{\epsilon }$ are the forward/backward discrete *y*-derivatives. The Toda equation corresponds to the deformed non-locality $w=\partial_{x}^{-1}\frac{T-1}{\epsilon }u$, while the Hamiltonian operator ∂_*x*_ and the Hamiltonian density *h* = e^*w*^ stay the same. Indeed, we have

$$\frac{\delta H}{\delta u}=\partial_{x}^{-1}\left(\frac{1-T^{-1}}{\epsilon }\right){\text{e}}^{w},$$

so that

$$u_{t}=\partial_{x}\frac{\delta H}{\delta u}=\left(\frac{1-T^{-1}}{\epsilon }\right){\text{e}}^{w},$$

as required.

## Data accessibility

This article has no additional data.

## Appendix A. Dispersionless Lax pair for *h* = *σ*(*u*) e^*w*^

Here we prove that expression (2.13),

$$G(p,u)=\ln\sigma (\lambda (p-u))+\epsilon \ln\sigma (\lambda (p-\epsilon u))+\epsilon^{2}\ln\sigma (\lambda (p-\epsilon^{2}u)),$$

where $\epsilon ={\text{e}}^{2\pi i/3}=-(1/2)+i(\sqrt{3}/2)$ and $\lambda =i/\sqrt{3}$, solves equations (2.12),

$$G_{p}=\frac{G_{uu}}{G_{u}}-\zeta (u),\;G_{uuu}G_{u}-2G_{uu}^{2}+2℘(u)G_{u}^{2}=0.$$

In what follows we will use the addition formula

A 1 $$\zeta (u+v)=\zeta (u)+\zeta (v)+\frac{1}{2}\frac{{℘}^{\prime }(u)-{℘}^{\prime }(v)}{℘(u)-℘(v)}.$$

We will also need the following fact.

Proposition A.3. *In the equianharmonic case, the Weiesrtrass functions satisfy the identity*

A 2 $$\lambda \frac{{℘}^{\prime }(\lambda u)}{℘(\lambda u)}+3\lambda \zeta (\lambda u)-\zeta (u)=0,\;\lambda =\frac{i}{\sqrt{3}}.$$

Proof. Using the standard expansions

$$\zeta (z)=\frac{1}{z}-\frac{g_{3}}{140}z^{5}-\ldots ,\;℘(z)=\frac{1}{z^{2}}+\frac{g_{3}}{28}z^{4}+\ldots ,$$

one can show that formula (A 2) holds to high order in *z* for the specific parameter value $\lambda =\frac{i}{\sqrt{3}}$. Therefore, it is sufficient to establish the differentiated (by *u*) identity (A 2), namely,

A 3 $$-\lambda^{2}℘(\lambda u)+\frac{\lambda^{2}g_{3}}{{℘}^{2}(\lambda u)}+℘(u)=0,$$

where we have used ℘ ″ = 6℘^2^ and ℘ ′^2^ = 4℘^3^ − *g*_3_. Explicitly, (A 3) reads

$$℘\left(\frac{iu}{\sqrt{3}}\right)-\frac{g_{3}}{{℘}^{2}(iu/\sqrt{3})}+3℘(u)=0.$$

Setting $u=i\sqrt{3}v$ we obtain

A 4 $$℘(v)-\frac{g_{3}}{{℘}^{2}(v)}+3℘(i\sqrt{3}v)=0.$$

Thus, it is sufficient to establish (A 4). Formulae of this kind appear in the context of complex multiplication for elliptic curves with extra symmetry. Let us begin with the standard invariance properties of the equianharmonic *ζ*-function:

$$\zeta (\epsilon z)=\epsilon^{2}\zeta (z),\;\zeta (\epsilon^{2}z)=\epsilon \zeta (z);$$

here $\epsilon ={\text{e}}^{2\pi i/3}=-(1/2)+i(\sqrt{3}/2)$ is the cubic root of unity. Setting *z* = 2*v* this gives

$$\zeta (-v+i\sqrt{3}v)=\epsilon^{2}\zeta (2v),\;\zeta (-v-i\sqrt{3}v)=\epsilon \zeta (2v).$$

Using the addition formula (A 1) one can rewrite these relations in the form

$$-\zeta (v)+\zeta (i\sqrt{3}v)+\frac{1}{2}\frac{-{℘}^{\prime }(v)-{℘}^{\prime }(i\sqrt{3}v)}{℘(v)-℘(i\sqrt{3}v)}=\epsilon^{2}\zeta (2v)$$

and

$$-\zeta (v)-\zeta (i\sqrt{3}v)+\frac{1}{2}\frac{-{℘}^{\prime }(v)+{℘}^{\prime }(i\sqrt{3}v)}{℘(v)-℘(i\sqrt{3}v)}=\epsilon \zeta (2v),$$

respectively. Adding there relations together (and keeping in mind that 1 + *ϵ* + *ϵ*^2^ = 0) we obtain

$$-2\zeta (v)-\frac{{℘}^{\prime }(v)}{℘(v)-℘(i\sqrt{3}v)}+\zeta (2v)=0.$$

Using the duplication formula *ζ*(2*v*) = 2*ζ*(*v*) + (3℘^2^(*v*)/℘ ′(*v*)), this simplifies to

$$-\frac{{℘}^{\prime }(v)}{℘(v)-℘(i\sqrt{3}v)}+\frac{3{℘}^{2}(v)}{{℘}^{\prime }(v)}=0,$$

which is equivalent to (A 4) via ℘ ′^2^ = 4℘^3^ − *g*_3_. □

Proposition A.4. *Expression (2.13) solves equations (2.12)*.

Proof. Computation of partial derivatives of *G*(*p*, *u*) gives

$$\begin{array}{rl} G_{p} & =\lambda \zeta (\lambda (p-u))+\lambda \epsilon \zeta (\lambda (p-\epsilon u))+\lambda \epsilon^{2}\zeta (\lambda (p-\epsilon^{2}u))\\ G_{u} & =-\lambda \zeta (\lambda (p-u))-\lambda \epsilon^{2}\zeta (\lambda (p-\epsilon u))-\lambda \epsilon \zeta (\lambda (p-\epsilon^{2}u)). \end{array}$$

Using the addition formula (A 1), the identity 1 + *ϵ* + *ϵ*^2^ = 0, and the invariance

$$\begin{array}{r} \zeta (\epsilon z)=\epsilon^{2}\zeta (z),\;\zeta (\epsilon^{2}z)=\epsilon \zeta (z),\\ ℘(\epsilon z)=\epsilon ℘(z),\;℘(\epsilon^{2}z)=\epsilon^{2}℘(z),\\ {℘}^{\prime }(\epsilon z)=℘(z),\;{℘}^{\prime }(\epsilon^{2}z)=℘(z), \end{array}$$

we obtain:

$$\begin{array}{rl} & \frac{1}{\lambda }G_{p}=\zeta (\lambda (p-u))+\epsilon \zeta (\lambda (p-\epsilon u))+\epsilon^{2}\zeta (\lambda (p-\epsilon^{2}u))\\ & =\zeta (\lambda p)-\zeta (\lambda u)+\frac{1}{2}\frac{{℘}^{\prime }(\lambda p)+{℘}^{\prime }(\lambda u)}{℘(\lambda p)-℘(\lambda u)}+\epsilon \left(\zeta (\lambda p)-\epsilon^{2}\zeta (\lambda u)+\frac{1}{2}\frac{{℘}^{\prime }(\lambda p)+{℘}^{\prime }(\lambda u)}{℘(\lambda p)-\epsilon ℘(\lambda u)}\right)\\ & \;+\epsilon^{2}\left(\zeta (\lambda p)-\epsilon \zeta (\lambda u)+\frac{1}{2}\frac{{℘}^{\prime }(\lambda p)+{℘}^{\prime }(\lambda u)}{℘(\lambda p)-\epsilon^{2}℘(\lambda u)}\right)\\ & =-3\zeta (\lambda u)+\frac{{℘}^{\prime }(\lambda p)+{℘}^{\prime }(\lambda u)}{2}\left(\frac{1}{℘(\lambda p)-℘(\lambda u)}+\frac{\epsilon }{℘(\lambda p)-\epsilon ℘(\lambda u)}+\frac{\epsilon^{2}}{℘(\lambda p)-\epsilon^{2}℘(\lambda u)}\right)\\ & =-3\zeta (\lambda u)+\frac{{℘}^{\prime }(\lambda p)+{℘}^{\prime }(\lambda u)}{2}\frac{3{℘}^{2}(\lambda u)}{{℘}^{3}(\lambda p)-{℘}^{3}(\lambda u)}\\ & =-3\zeta (\lambda u)+\frac{{℘}^{\prime }(\lambda p)+{℘}^{\prime }(\lambda u)}{2}\frac{12{℘}^{2}(\lambda u)}{{℘}^{\prime 2}(\lambda p)-{℘}^{\prime 2}(\lambda u)}\\ & =-3\zeta (\lambda u)+\frac{6{℘}^{2}(\lambda u)}{{℘}^{\prime }(\lambda p)-{℘}^{\prime }(\lambda u)}. \end{array}$$

A similar calculation gives:

$$\begin{array}{rl} & -\frac{1}{\lambda }G_{u}=\zeta (\lambda (p-u))+\epsilon^{2}\zeta (\lambda (p-\epsilon u))+\epsilon \zeta (\lambda (p-\epsilon^{2}u))\\ & =\zeta (\lambda p)-\zeta (\lambda u)+\frac{1}{2}\frac{{℘}^{\prime }(\lambda p)+{℘}^{\prime }(\lambda u)}{℘(\lambda p)-℘(\lambda u)}\\ & \;+\epsilon^{2}\left(\zeta (\lambda p)-\epsilon^{2}\zeta (\lambda u)+\frac{1}{2}\frac{{℘}^{\prime }(\lambda p)+{℘}^{\prime }(\lambda u)}{℘(\lambda p)-\epsilon ℘(\lambda u)}\right)\\ & \;+\epsilon \left(\zeta (\lambda p)-\epsilon \zeta (\lambda u)+\frac{1}{2}\frac{{℘}^{\prime }(\lambda p)+{℘}^{\prime }(\lambda u)}{℘(\lambda p)-\epsilon^{2}℘(\lambda u)}\right)\\ & =\frac{{℘}^{\prime }(\lambda p)+{℘}^{\prime }(\lambda u)}{2}\left(\frac{1}{℘(\lambda p)-℘(\lambda u)}+\frac{\epsilon^{2}}{℘(\lambda p)-\epsilon ℘(\lambda u)}+\frac{\epsilon }{℘(\lambda p)-\epsilon^{2}℘(\lambda u)}\right)\\ & =\frac{{℘}^{\prime }(\lambda p)+{℘}^{\prime }(\lambda u)}{2}\frac{3℘(\lambda p)℘(\lambda u)}{{℘}^{3}(\lambda p)-{℘}^{3}(\lambda u)}\\ & =\frac{{℘}^{\prime }(\lambda p)+{℘}^{\prime }(\lambda u)}{2}\frac{12℘(\lambda p)℘(\lambda u)}{{℘}^{\prime 2}(\lambda p)-{℘}^{\prime 2}(\lambda u)}\\ & =\frac{6℘(\lambda p)℘(\lambda u)}{{℘}^{\prime }(\lambda p)-{℘}^{\prime }(\lambda u)}. \end{array}$$

To summarize, we have:

$$G_{p}=-3\lambda \zeta (\lambda u)+\frac{6\lambda {℘}^{2}(\lambda u)}{{℘}^{\prime }(\lambda p)-{℘}^{\prime }(\lambda u)},\;G_{u}=-\frac{6\lambda ℘(\lambda p)℘(\lambda u)}{{℘}^{\prime }(\lambda p)-{℘}^{\prime }(\lambda u)}.$$

This gives

A 5 $$\frac{G_{uu}}{G_{u}}={\left(\ln G_{u}\right)}_{u}=\lambda \frac{{℘}^{\prime }(\lambda u)}{℘(\lambda u)}+\frac{6\lambda {℘}^{2}(\lambda u)}{{℘}^{\prime }(\lambda p)-{℘}^{\prime }(\lambda u)},$$

and the first equation (2.12), $G_{p}=\frac{G_{uu}}{G_{u}}-\zeta (u)$, is satisfied identically due to (A 2). Finally, the second equation (2.12), $G_{uuu}G_{u}-2G_{uu}^{2}+2℘(u)G_{u}^{2}=0$, which can be written in the equivalent form

$$-{\left(\frac{G_{uu}}{G_{u}}\right)}_{u}+{\left(\frac{G_{uu}}{G_{u}}\right)}^{2}=2℘(u),$$

is satisfied identically due to (A 5) and (A 3). □
